# Facile Fabrication of Superhydrophobic Surface from Fluorinated POSS Acrylate Copolymer via One-Step Breath Figure Method and Its Anti-Corrosion Property

**DOI:** 10.3390/polym11121953

**Published:** 2019-11-28

**Authors:** Meng Liu, Xiaochen Zhang, Dong Wang, Jiaji Cheng, Xiujiang Pang, Wenjuan Qu, Chunxu Li, Shaoxiang Li

**Affiliations:** 1 Shandong Engineering Research Center for Marine Environment Corrosion and Safety Protection, College of Environment and Safety Engineering, Qingdao University of Science and Technology, Qingdao 266042, China; liumengqust@163.com (M.L.); zxcse7en@126.com (X.Z.); wd_charrel@163.com (D.W.); cjj_cumt@163.com (J.C.); quwenjuan2016@163.com (W.Q.); 2 Shandong Engineering Technology Research Center for Advanced Coating, Qingdao University of Science and Technology, Qingdao 266042, China; 3 State Key Laboratory Base of Eco-chemical Engineering, College of Chemistry and Molecular Engineering, Qingdao University of Science and Technology, Qingdao 266042, China; xiujiangpang@qust.edu.cn; 4 ASTUTE 2020 in Future Manufacturing Research Institute, College of Engineering, Swansea University, Wales SA18EN, UK

**Keywords:** fluorinated POSS acrylic copolymers, superhydrophobic coating, one-step breath figure method, chemical constitution, morphology, hydrophobicity, anticorrosion performance

## Abstract

Novel fluorinated polyhedral oligomeric silsesquioxane (POSS) acrylic copolymers were synthesized by the radical solution polymerization. The superhydrophobic coating was prepared using a one-step breath figure method. Chemical constitution, morphology, hydrophobicity, and anticorrosion ability of as-prepared coatings were investigated by the corresponding equipment. The addition of proper fluorinated POSS can synchronously promote the formation of the micro-nano convex structure and the enrichment of fluorinated groups on the surface. Compared to commercial acrylic coating, the fluorinated POSS coating presented enhanced anticorrosion performance. The impedance was the highest and the corrosion current density was the lowest for superhydrophobic coating with 25 wt % fluorinated POSS.

## 1. Introduction

Researches on superhydrophobic surfaces with a water contact angle (WCA) of more than 150° [1] have been widely investigated due to its great potential application in the areas of corrosion protection [2,3], self-cleaning [4,5], oil/water separation [6,7], etc. Water repellency is dependent on the microstructure and chemistry of the surfaces. For a flat surface, theoretical calculation shows that the highest contact angle of water could reach 120° [8,9]. It is well known that creating appropriate roughness on a hydrophobic surface or modifying a rough one by low-surface-energy materials are two main routes to obtain super-hydrophobic surfaces [10].

The porous structure is a typical topological structure with greater roughness than the flat film. The breath figures (BF) method is a self-assembly template technology and has been widely employed to form porous polymer films because of a reduction in the production time and costs [11]. The water contact angles of different honeycomb porous films fabricated with PS [12], acrylate copolymers [13] and other polymers via BF method range from 110° to 130°. According to the reported literatures, there are three strategies to obtain the superhydrophobic property from the honeycomb porous structure fabricated by the BF method. The first one is peeling off the top layer to provoke the fracture of the pillars in pins and form a pincushion-like structure. Yabu et al. [14] prepared fluorinated acrylate copolymer films with honeycomb porous structure using the BF method, and the superhydrophobic behaviour was achieved by surface peeling with a maximum contact angle of 170°. Super-hydrophobic nano-needle arrays were simply fabricated by removing the top portion of the honeycomb films and the maximum water contact angle was 150° [15]. The second one is implementing another surface texturing on the ordered honeycomb surfaces. Brown et al. [16] introduced cross-linking and surface texturing by CF**_4_** plasmachemical fluorination on the polybutadiene honeycomb surfaces with the contact angle increasing to 170°. The porous PS/SiO**_2_** films were furthermore modified by chemical vapour deposition (CVD) of chlorotrimethylsilane on the film surface under 100 °C for 2 h to achieve the superhydrophobic behaviour [12]. Reducing the pore size and rim width under controllable conditions is the third viable strategy to obtain the desired film. A superhydrophobic honeycomb-patterned film with 300 nm pores and 200 nm rim was prepared with the wet film thickness of 100 μm on a glass substrate modified by ultra-ozone treatment, immersion in perfluorooctylchlorosilane solution and baking at 120 °C [17]. However, these strategies for fabrication of porous structure with superhydrophobic behaviour contain severe conditions, such as surface peeling, second surface texturing, and complex multi-step processing, limiting their practical application.

Over the past decades, inspired from nature self-cleaning surface, a lot of surface structures have been proposed to be superhydrophobic, such as hierarchical stripes [18], hemispheroid [19], spikes [20], interlaced Noodles or refibres [21,22,23] and random stacking nano-particle [24,25,26,27]. The characteristics of the convex sections contacting with water droplets on these structures almost are nano-micro hierarchical and discrete. Meanwhile, the discreteness spikes pattern structure was proved to be highly hydrophobic in spite of low roughness, compared with shallow cavities and stripes [28]. Therefore, a hierarchical and discrete rim structure of the porous film prepared via controllable BF method might further enhance the water repellency. In the traditional BF process, the formation of honeycomb structures required that the condensed water droplets can be stabilized by an end-functional polymer or by particles added to the polymer solution [29]. Coalesce action occurred when the condensed water droplets were not very stable during the BF process [30]. However, it is difficult to control the coalesce action to break the rim into discrete structure and there are no reports related to the formation of hierarchical and discrete rim structure depending on the fusion of water droplets during the BF method.

Polyhedral oligomeric silsesquioxane (POSS), the smallest hybrid particle of silica [31], is a cube-octameric molecule with an inner inorganic Si-O-Si structure that is externally covered by organic substituents [32,33]. In the past decades, POSS-incorporated polymer materials have attracted increasing interest due to the using in the construction of the multifunctional nanohybrids and nanocomposites with tunable hierarchical structures and unparalleled properties [34,35,36]. Several different POSS fluorinated acrylates were synthesized and applied for hydrophobic honeycomb-patterned porous films through the breath figure (BF) method [37,38]. It has been proved that the addition of POSS was propitious to from the honeycomb structures during the BF process. However, the reported POSS fluorinated acrylates are incapable of controlling the coalesce action of water droplets during the BF process.

Here, a fluorinated POSS with seven fluorine-containing groups connected with the Si atom was synthesized and used to tune the amphipathic property of POSS-based polymer. Then, we proposed a one-step BF process for the fabrication of a superhydrophobic surface with the fluorinated POSS-acrylate random copolymer solution. The hierarchical and discrete convex structure of the superhydrophobic surface was constructed by taking advantage of the self-assembly of the fluorinated POSS in the forming process of BF pattern. The variation of surface topographies and wettability as a function of fluorinated POSS content was investigated. The electrochemical behaviours of the fabricated superhydrophobic surface were discussed in detail. The resultant superhydrophobic surface exhibits a remarkable marine anticorrosion ability. We believe this one step BF process may supply great convenience for constructing the superhydrophobic surface and extending its potential functional applications.

## 2. Materials and Methods

### 2.1. Materials 

3,3,3-trifluoropropyl trimethoxysilane (TFP-TMS) and 2-Perfluorohexyl ethyl methacrylate (PFHEMA) were purchased from Qinba Chemical Co., Ltd., Shanghai, China. Methylacrylatepropyl trichlorosilane (MAPTS) was supplied by Gelest. Methyl methacrylate (MMA) and butyl acrylate (BA) were obtained from BASF Chemical Co., Ltd., Tianjin, China. Acrylic acid (AA) was purchased from Damao Chemical Reagent Factory, Tianjin, China. Azobisisobutyronitrile (AIBN) was supplied by Kemiou Chemical Reagent Co., Ltd., Tianjin, China. Triethylamine, tetrahydrofuran, butyl acetate, xylene, 1,1,2-trifluorotrichloroethane, chloroform and sodium hydroxide were obtained from China National Medicines Co., Ltd. Calcium hydride was purchased from Aladdin Reagent Co., Ltd., Shanghai, China.

### 2.2. Synthesis of Methylacrylatepropyl Hepta(3,3,3-trifluoropropyl) POSS (7F-MAP-POSS)

**Figure 1:**
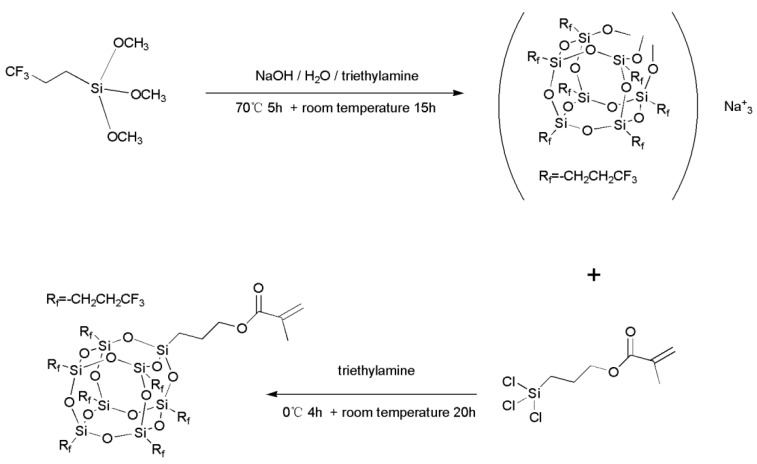
Synthesis of 7F-Methylacrylatepropyl (MAP)-polyhedral oligomeric silsesquioxane (POSS).

Methylacrylatepropyl hepta(3,3,3-trifluoropropyl) POSS was prepared through a typically two-step reaction (Figure 1). First, hepta(3,3,3-trifluoropropyl) tricycloheptasiloxane trisodium silanolate, 7F-T_7_-(ONa)_3_, was synthesized using the method reported by Fukuda et al. [39]. 3,3,3-trifluoropropyl trimethoxysilane (50.0 g, 0.23 mol), THF (250 mL), deionized water (5.25 g, 0.29 mol) and sodium hydroxide (3.95 g, 0.1 mol) were charged to a flask equipped with a reflux condenser and a magnetic stirrer. After refluxed in an oil bath at 70 °C for 5 h, the reactive system was cooled down to room temperature and held for 15 h with vigorous stirring. All the volatile components were removed by rotary evaporation and the obtained white solids were dried at 40 °C in a vacuum for 24 h to yield the 7F-T_7_-(ONa)_3_ as a white powder (38 g) in a quantitative yield. Second, 7F-MAP-POSS was prepared by following the method reported by Ke Zeng et al. [40]. 7F-T_7_-(ONa)_3_ (10.0 g, 8.8 mmol) and triethylamine (1.3 mL, 8.8 mmol) were charged to a flask equipped with a magnetic stirrer, 200 mL dry THF were added with vigorous stirring. The system was immersed into an ice-water bath and purged using highly pure nitrogen for 1 h. Then, Methylacrylatepropyl trichlorosilane (2.91 g, 10.56 mmol) dissolved in 20 mL dry THF was slowly dropped within 30 min. After immersed in the ice-water bath for 4 h, the reactive system was taken out and held at room temperature for 20 h with vigorous stirring. The resultant precipitate was removed by centrifugal separation, and the supernatant fluid was concentrated by a rotary evaporator to obtain a crude product. The obtained 7F-MAP-POSS was washed 3 times with 50 mL methanol and dried in vacuum at 40 °C for 24 h**,** and 7.6 g of product was obtained. ^29^Si NMR (CDCl_3_, 400MHz) δ −67.61 (s, 1Si, Si–CH_2_CH_2_CH_2_-), −69.94 (s, 3Si, Si–CH_2_CH_2_CF_3_), −69.44 (s, 1Si, Si–CH_2_CH_2_CF_3_), and −68.95 (s, 3Si, Si–CH_2_CH_2_CF_3_). 1H NMR (CDCl_3_, 400MHz) δ 0.62 (s, 2H, Si–CH_2_CH_2_CH_2_O–), 0.92 (m, 2H, Si–CH_2_CH_2_CF_3_), 1.18 (s, 2H, Si–CH_2_CH_2_CH_2_O–), 1.86 (s, CH_3_-C), 6.03 (s, 1H, C=CH_2_), 2.13 (s, 2H, –CH_2_CF_3_), 4.06 (s, 2H, Si–CH_2_CH_2_CH_2_O–), 6.03 (s, 1H, C=CH_2_), 5.50 (s, 1H, C=CH_2_).

### 2.3. Synthesis of the 7F-MAP-POSS-Acrylate Copolymers

**Figure 2:**
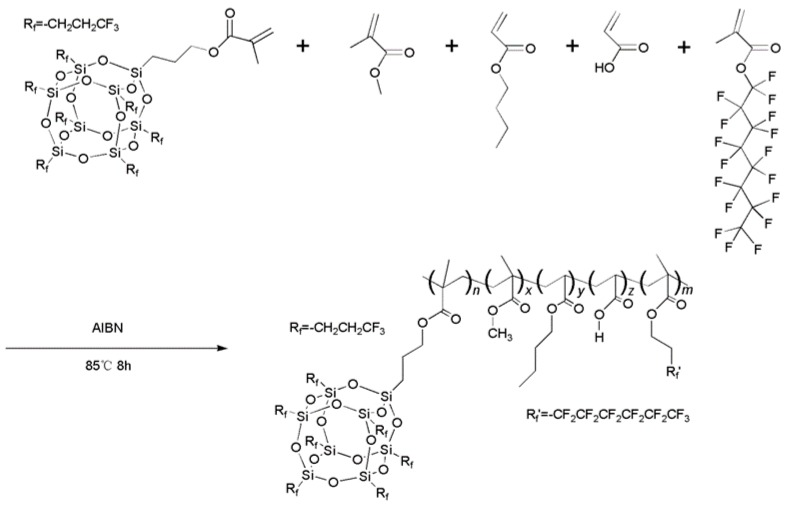
Synthesis of 7F-MAP-POSS-Acrylate copolymers.

The 7F-MAP-POSS-Acrylate copolymers were prepared by the radical solution polymerization (Figure 2). The reaction solvent (15 g), butyl acetate/dimethylbenzene (*v*/*v*, 1:1), was placed into a flask equipped with a reflux condenser and a magnetic stirrer at 85 °C. Then the system was purged using highly pure nitrogen for 30 min. A mixture of MMA (4.4 g), BA (1.45 g), AA (0.02 g), AIBN (0.06 g) and the reaction solvent (1 g) was slowly dropped within 1h. After about 1h, the amount of the solution (1 g), trifluorotrichloroethane (3 g), 2-Perfluorooctane ethyl methacrylate (1.5 g) and AIBN (0.02 g) was added into the flask. After about 1h, the mixture of 7F-MAP-POSS (0, 0.82, 1.84 and 2.46 g for Samples 1–4 correspondingly), the reaction solvent (0, 3.28, 7.36 and 9.84 g for Samples 1–4 correspondingly) and AIBN (0, 0.001, 0.018, 0.025 g for Samples 1–4 correspondingly) was added into the flask. After about 1 h, AIBN (0.015, 0.016, 0.018 and 0.020g for Samples 1–4 correspondingly) and the reaction solvent (2 g) were added into the flask. After being stirred for another 4 h, the reactants were concentrated by a rotary evaporator. The resulting production was precipitated in cool methanol and dried under vacuum at 40 °C for 24 h. The obtained samples 1–4 (6.3, 6.9, 7.8 and 8.4g) were named as FAC, FPOSS10-FAC, FPOSS20-FAC and FPOSS25-FAC with the 7F-MAP-POSS content of 0, 10, 20 and 25 wt %, respectively.

### 2.4. Preparation of the Superhydrophobic 7F-MAP-POSS-Acrylate Copolymer Coatings

**Figure 3:**
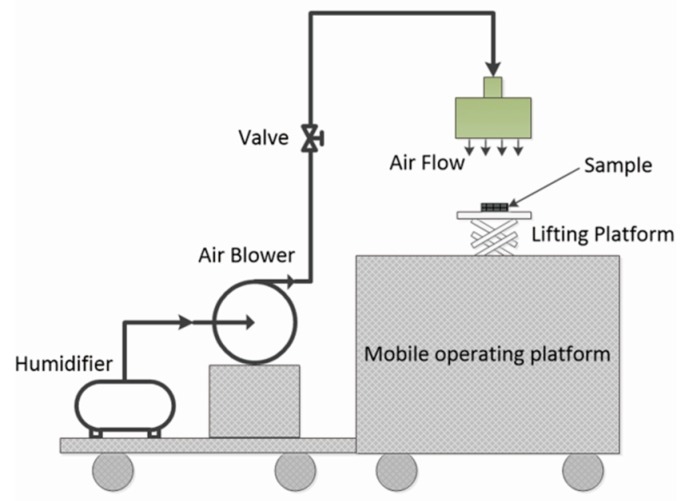
The assembled equipment for breath figure method.

Firstly, the Q235 steel sheets were cleaned by successively wiping the surface with absolute ethanol and chloroform and dried under atmosphere. Then, 7F-MAP-POSS-Acrylate copolymer solutions of fixed volume in chloroform were directly cast onto the Q235 steel substrates. The wet coatings were blown by the humid air (relative humidity ~85% at room temperature) containing micro ultrasonic atomized water droplets using the assembled equipment shown in Figure 3. The flow velocity was approximately 0.8 m/s. The solidified film was then dried at room temperature.

### 2.5. Characterizations

Fourier transform infrared (FT-IR) spectrum of F-POSS and F-POSS-Acrylate copolymer were measured by a TENSOR 27 FT-IR spectrometer (Bruker, Karlsruhe, Germany) from 400 to 4000 cm^−1^. The samples were prepared by following the KBr pellet method. ^29^Si NMR and 1H NMR spectrum of 7F-MAP-POSS before and after copolymerization process were recorded on a 500MHz AVANCE III spectrometer (Bruker, Karlsruhe, Germany) using CDCl_3_ as a solvent. A gel permeation chromatography (GPC) system (Agilent, Palo Alto, CA, USA) was calibrated with narrow molecular weight polystyrene standards. The resulting calibration curves were used to estimate the molecular weights and polydispersity (PD) of 7F-MAP-POSS-Acrylate copolymers. THF was introduced as the mobile phase at a flow rate of 1.0 mL/min. The concentrations of samples were 5.0 mg × mL^−1^, and the injection volume was 100 μL for each analysis. The columns and detector were maintained at 40 °C. Contact angles (CA) were measured using SL200KS contact angle analyzer (KINO, Boston, MA, USA) that had a CCD camera equipped for image capture.

Scanning electron microscopy (SEM) images and energy dispersive spectrum (EDS) were taken using a JSM-6700F scanning electron microscope (JEOL, Tokyo, Japanese). The surface morphology of the films was observed after gold was sputtered onto the surfaces.

### 2.6. Electrochemical Tests

All the electrochemical tests were performed with AMETEK PARSTAT 4000 by immersing the specimens into a 3.5% NaCl aqueous solution. In a typical process, a standard three-electrode system equipped with a saturated calomel reference electrode, Pt plate as a counter electrode and specimen as the working electrode were employed. The electrochemical impedance spectroscopy (EIS) was carried out at open circuit potential (OCP) in the frequency range of 10^5^ Hz–10^−2^ Hz. Prior to electrochemical tests, the specimens with 1 cm^2^ area exposure were immersed in 3.5% NaCl aqueous solution for more than 30 min to stabilize the system. All of the EIS data were fitted and analysed through ZSimpWin software. The polarization curves were recorded at a scan rate of 0.5 mV/s in the applied potential range from −250 to 250 mV. Corrosion potential, E_corr_, current and i_corr_, were derived from the potentiodynamic polarization curves after Tafel extrapolation.

## 3. Results and Discussion

### 3.1. Chemical Characterization

**Figure 4:**
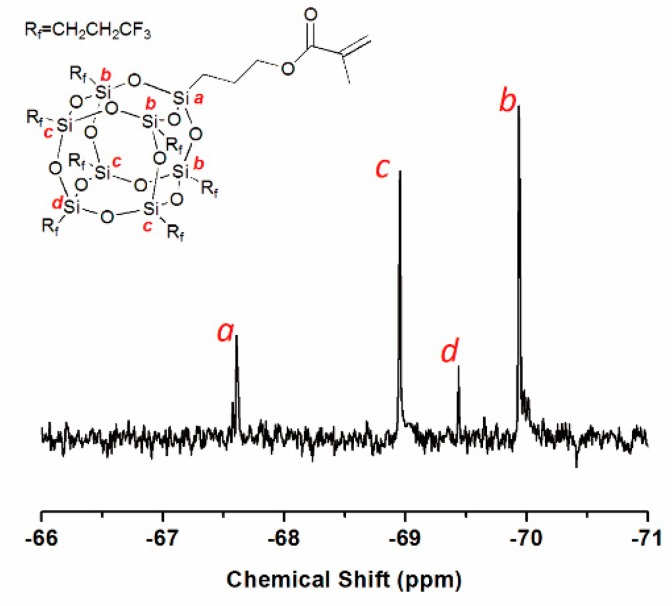
^29^Si NMR spectrum of 7F-MAP-POSS.

The chemical composite and structure of 7F-MAP-POSS and 7F-MAP-POSS acrylate copolymers were characterized by NMR and FTIR. As shown in Figure 4, the ^29^Si NMR spectroscopy was used to demonstrate the formation of an octameric silsesquioxane cage. The resonance at −67.61 ppm is assignable to the corner silicon atom connected to methylacrylatepropyl group [41], while the signals of resonance at −69.94, −69.44 and −68.95 ppm are ascribed to the other silicon nucleus of silsesquioxane with different distances from the corner silicon atom. In terms of the ratio of integration intensity of the resonance [28], it is confirmed that the octameric silsesquioxane was formed.

**Figure 5:**
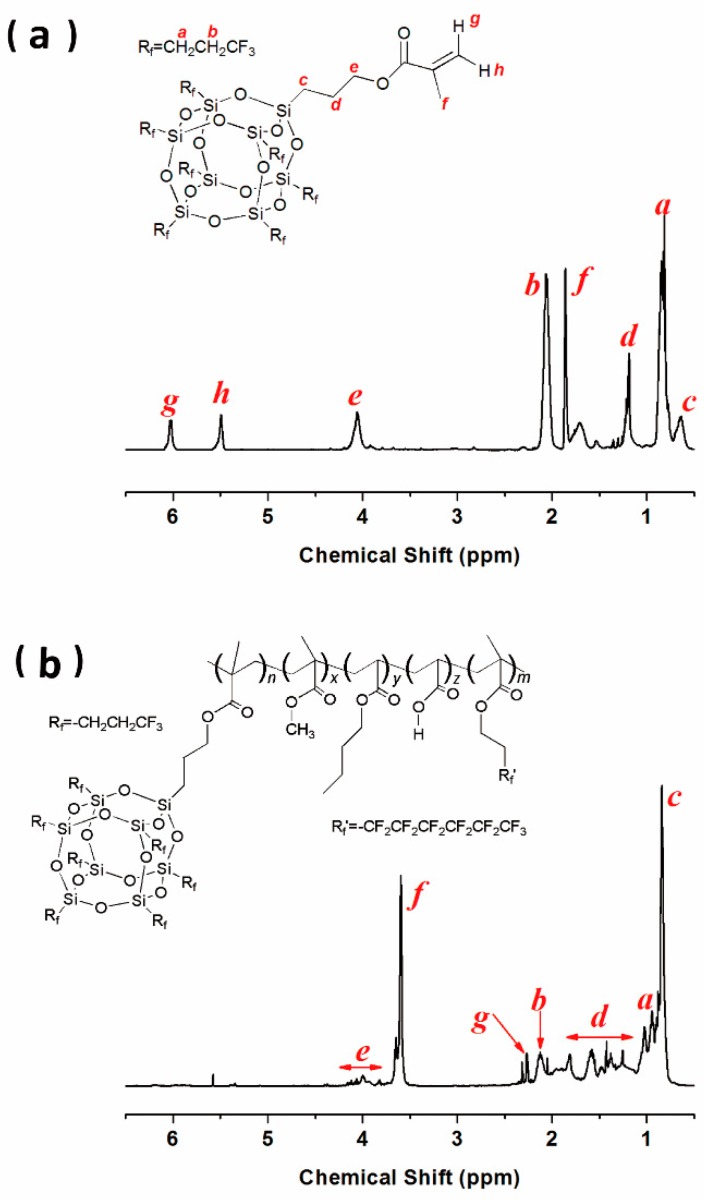
^1^H NMR spectra of 7F-MAP-POSS (**a**) and FPOSS25-FAC (**b**).

**Figure 6:**
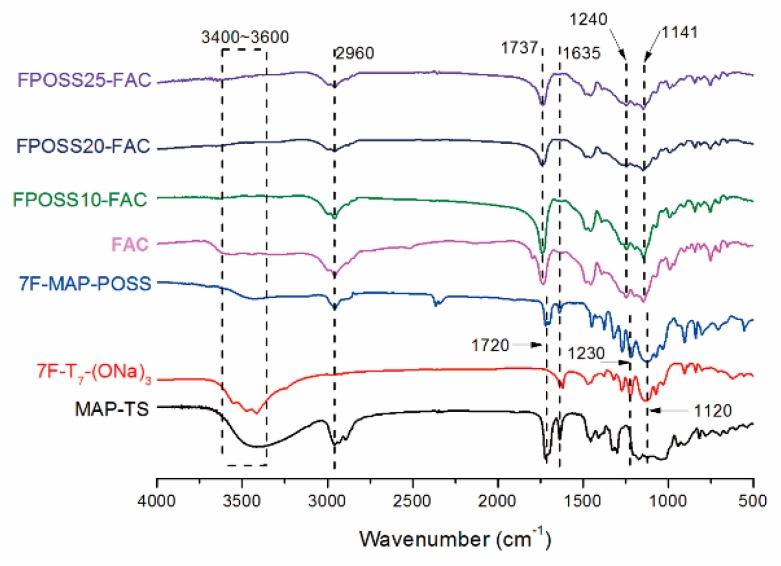
FT-IR spectra of MAP-TS, 7F-T7-(ONa)_3_, 7F-MAP-POSS, FAC, FPOSS10-FAC, FPOSS20-FAC and FPOSS25-FAC.

The chemical structure of 7F-MAP-POSS acrylate copolymer is confirmed by comparison of its 1H NMR spectrum in Figure 5B with the one of 7F-MAP-POSS in Figure 5A. Except for the typical resonance at 0.92 (–Si–CH_2_CH_2_CF_3_) and 2.13 (–CH_2_CF_3_) for 7F-MAP-POSS [42], the appearances of the methylene group at 3.62 ppm corresponding to CH_3_O– were found indicating the successful polymerization of MMA. The signal at 3.80–4.15 ppm from –CH_2_CH_2_O– and 2.26 ppm assigned to –CH_2_CH_2_–CF_2_– can confirm the successful polymerization of PFHEMA. The polymerization of BA can be proved by the signal at 3.80–4.15 ppm (–CH_2_CH_2_O–), 1.25–1.81 ppm (–CH_2_CH_2_–CH_2_–) and 0.84 ppm (–CH_3_). Unfortunately, the polymerization of AA cannot be confirmed from the ^1^H NMR spectrum because of the minimal addition. The FT-IR spectrum of MAP-TS, 7F-T_7_-(ONa)^3^, 7F-MAP-POSS and as-prepared copolymers were shown in Figure 6. In the curve of 7F-MAP-POSS, the peak at 1120 and 1230 cm^−1^ are attributed to Si-O-Si and C-F groups, respectively. The appearance of the MAP group in the structure of 7F-MAP-POSS can be proved by strong vibration bands at 1730 cm^−1^ (C=O) and 2960 cm^−1^ (CH_3_). Meanwhile, the peaks in the range of 3400–3600 cm^−1^ (OH) in spectra of MAP-TS and 7F-T_7_-(ONa)_3_ are disappeared in 7F-MAP-POSS due to the complete corner-capping reaction between MAP-TS and 7F-T7-(ONa)_3_. For the copolymers, the characteristic bands at 1737 cm^−1^ is assigned to the carbonyl group in MMA, PFHEMA, BA, AA and 7F-MAP-POSS units, while the bands at 1240 cm^−1^ are attributed to C-F group. The peak at 1141 cm^−1^ is due to the combination of stretching vibration of C-O-C and Si-O-Si. The NMR and FTIR analysis verified the successful synthesis of 7F-MAP-POSS and FPOSS-FACs.

On the other hand, the molecular weights and their polydispersity (PDI) of FAC and 7F-MAP-POSS-Acrylate copolymers are calculated from GPC curves (Table 1). With the increasing of 7F-MAP-POSS content, the molecular weights are increased from 28,215 g·mol^−1^ for FAC to 31,378 g·mol^−1^ for FPOSS20-FAC with a little wide PDI of 1.9–2.0. However, the molecular weights were decrease to 29,415 g·mol^−1^ for FPOSS25-FAC when the 7F-MAP-POSS. Therefore, the obtained results indicate that 7F-MAP-POSS is successfully reacted with the other acrylate monomers to form a 7F-MAP-POSS-Acrylate copolymer.

### 3.2. Morphology and Superhydrophobicity of the Superhydrophobic 7F-MAP-POSS-Acrylate Copolymer Coatings

**Figure 7:**
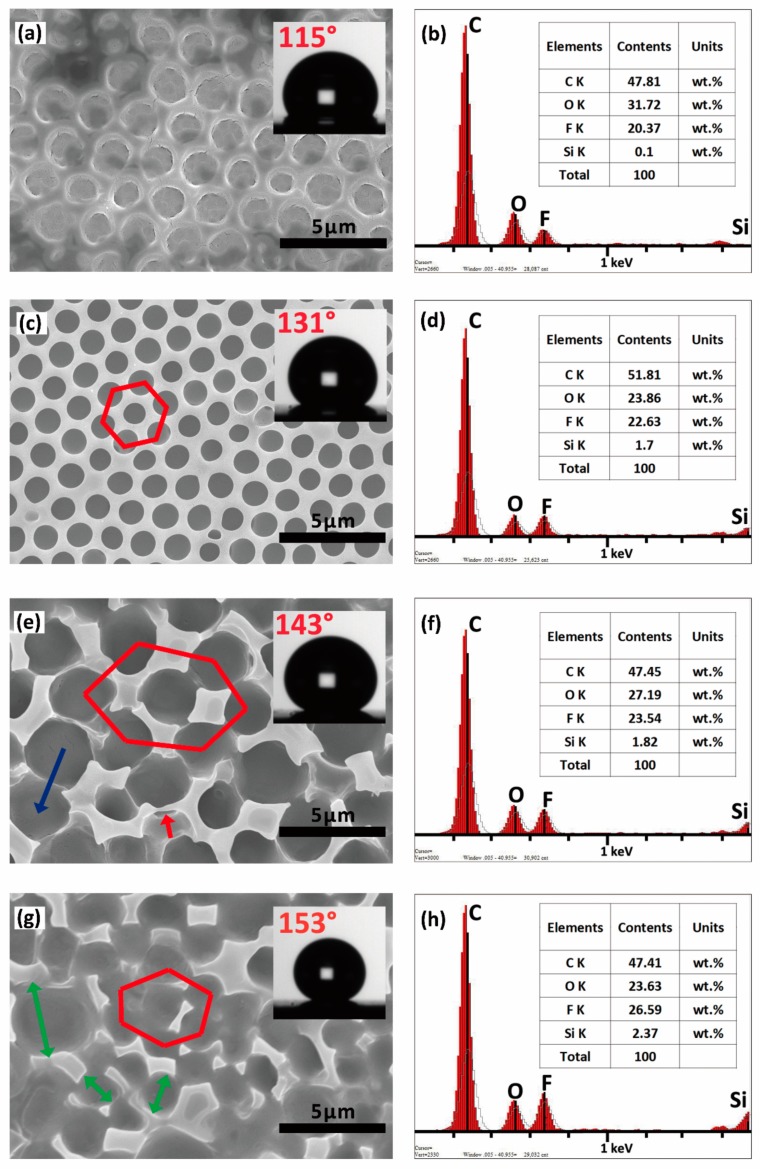
SEM images and energy dispersive spectrum (EDS) results of FAC (**a**,**b**), FPOSS10-FAC (**c**,**d**), FPOSS20-FAC (**e**,**f**) and FPOSS25-FAC (**g**,**h**).

The 7F-MAP-POSS-Acrylate copolymer coatings cast from chloroform under a flow of humid air were investigated via SEM. As shown in Figure 7a, the FAC coating without 7F-MAP-POSS showed a relatively ordered sunk structure, and the WCA value was the lowest at 115°. The highly ordered porous structure displayed in Figure 7c demonstrates that the use of 7F-MAP-POSS can assist the pore formation process from droplet nucleation through to chloroform evaporation and drying. The WCA value was about 131°, presenting a significantly hydrophobic property. The smaller surface tensions of FPOSS10-FAC solution due to the addition of fluorinated POSS means that during film formation, a bigger portion of the spherical water droplets may penetrate inside the solution before motion freezing and film hardening [43]. However, when the content of 7F-MAP-POSS increased up to 20 wt %, the increasing hydrophobicity of the copolymer derived from the fluorinated POSS would reduce the stability of condensed water droplets. Subsequently, the growth of water droplets would be enhanced and unstable, which led to the attenuation of the rim between two pores. An obvious coalescent of two water droplets begins at the middle of the rim as marked by the red arrows. Finally, a disorder and discrete convex rim structures were obtained as a result of the fusion indicated by two neighbouring holes sharing a sunken part of the pore wall, as marked by the blue arrow. The WCA increases up to 143° because of the reduced surface of FPOSS20-FAC film in contact with the water. As the content of 7F-MAP-POSS increased up to 25 wt %, the further enhanced hydrophobicity of the copolymer would encourage the pores fusion and provoke the more fracture of the rim with the formation of micro-nano convex structures shown in Figure 7g. The superhydrophobic film was obtained with the WCA increasing up to 153°.

**Figure 8:**
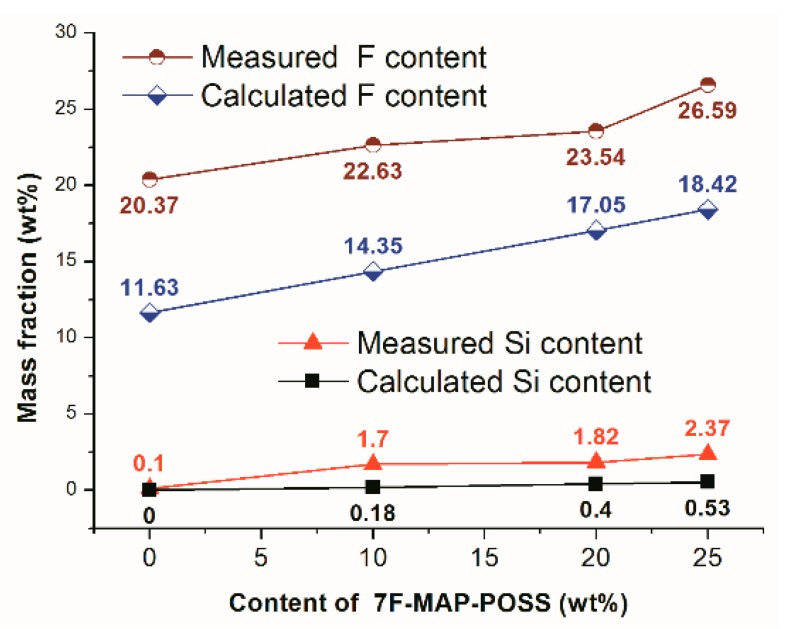
Theoretical and measured Si and F elements contents of as-prepared coatings.

EDS was performed to analyse the elemental chemistry of the as-prepared surfaces and the result was shown in Figure 7. From Figure 7b,d,f,h, we can see that the Si and F contents on the surfaces increase with the 7F-MAP-POSS content. Therefore, the increasing WCA values can be explained by the lower surface energy and the higher appropriate roughness. To investigate the migration behaviour of 7F-MAP-POSS, the theoretical contents of Si and F elements were calculated and compared with the measured values. As shown in Figure 8, the measured Si and F contents on the surfaces prepared from the 7F-MAP-POSS acrylate copolymers were higher than the calculated ones due to the migration and enrichment of PFHEMA and 7F-MAP-POSS onto the surface.

**Figure 9:**
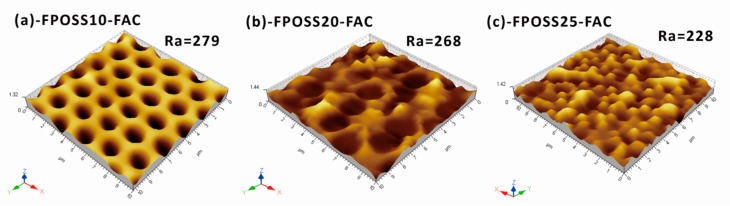
AFM morphologies and surface roughnesses of FPOSS10-FAC (**a**), FPOSS20-FAC (**b**) and FPOSS25-FAC (**c**).

**Figure 10:**
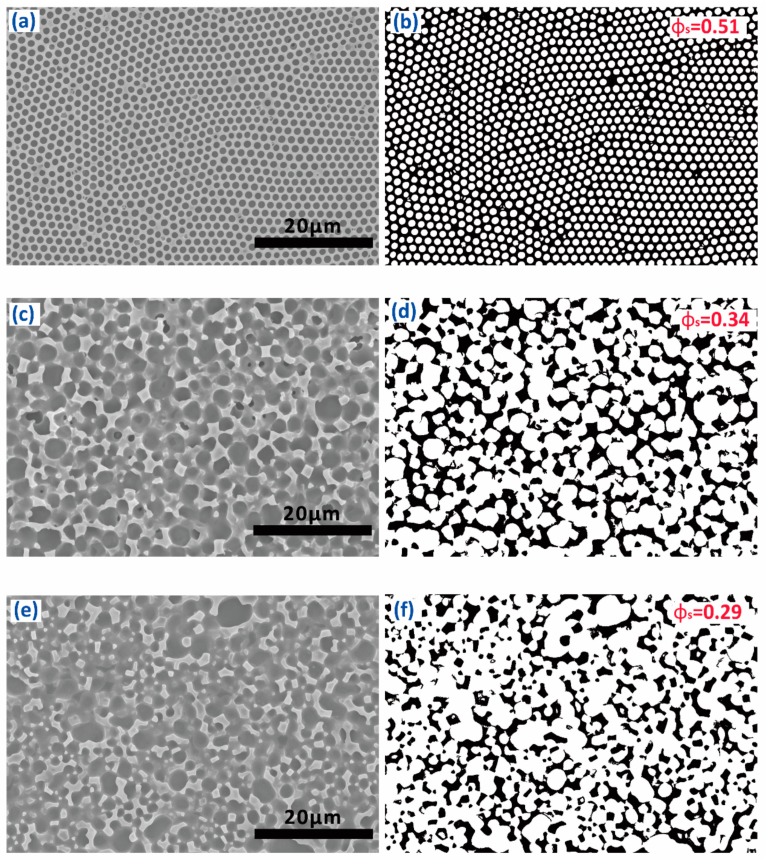
SEM images under 2000× magnification and corresponding grey analysis results of FPOSS10-FAC (**a**,**b**), FPOSS20-FAC (**c**,**d**) and FPOSS25-FAC (**e**,**f**).

**Figure 11:**
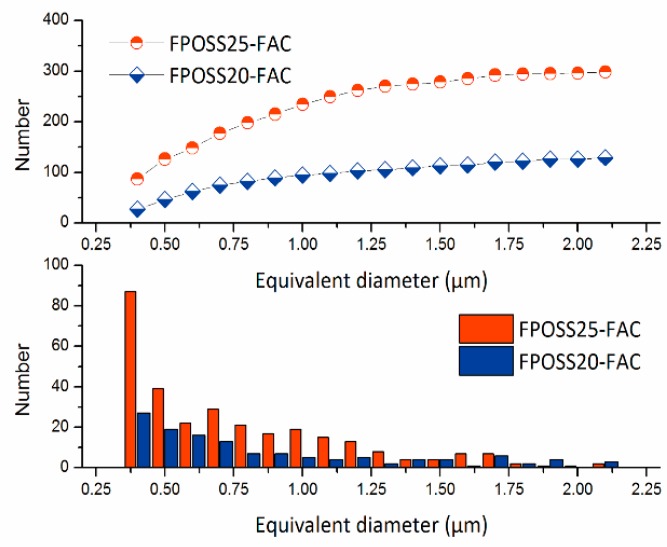
Equivalent diameter distribution of the convex structures of FPOSS20-FAC and FPOSS25-FAC.

To investigate the roughness of FPOSS-FAC film with different 7F-MAP-POSS contents, AFM was performed on the surfaces, and the results were shown in Figure 9. For the FPOSS10-FAC, the copolymer area was relatively smooth, and the Ra value was 279 nm by measurement. However, the Ra value decreased with the addition of 7F-MAP-POSS. When the 7F-MAP-POSS content increased to 25 wt %, the Ra value reduced to 228 nm and the copolymer area was rough. These results were inconsistent with the WCAs. The grey scale analysis method by imaging software was applied to the SEM images under 2000× magnification of the FPOSS-FAC film (Figure 10a,c,e). The convex sections of the surfaces were separated and displayed as black areas in Figure 10b,d,f. For FPOSS20-FAC and FPOSS25-FAC, the convex sections presented an obviously discrete distribution. If the convex sections were equivalent to the independent circular structure, the sizes and numbers of the discrete sections could be obtained using imaging software. As shown in Figure 11, the numbers of convex sections with the equivalent diameter less than 1.0 μm on FPOSS25-FAC film was larger than that for FPOSS20-FAC film and the total number was twice as many as the one for FPOSS20-FAC film. These results demonstrated that a remarkable micro-nano structure was fabricated by the one-step BF method from the FPOSS25-FAC.

**Figure 12:**
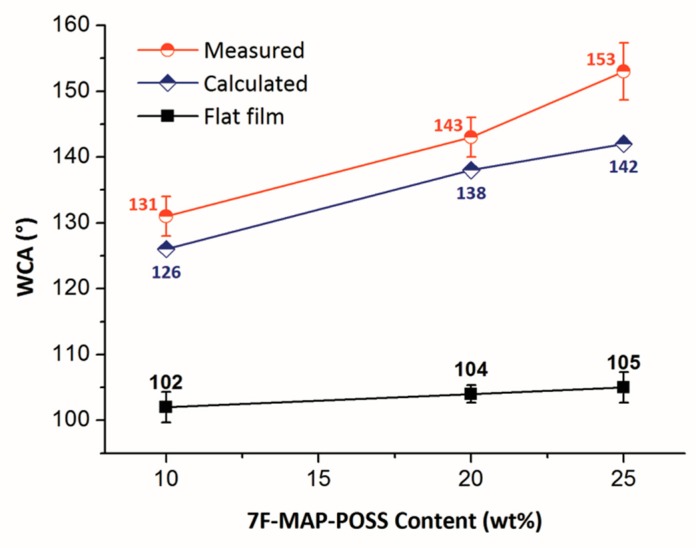
Theoretical and measured contact angles of FPOSS10-FAC, FPOSS20-FAC and FPOSS25-FAC.

According to Cassie’s law [44]:

$$\cos\theta^{*}={Ø}_{s}\left(1+\cos\theta_{e}\right)-1$$

where $\theta^{*}$ is the superficial contact angle; $\theta_{e}$ represents the contact angle of the flat film; ${Ø}_{s}$ denotes the fractional flat geometrical area of liquid–polymer interface beneath a water droplet. As shown in Figure 7, the surface area fractions of the films (${Ø}_{s}$) were calculated. By using the Cassie’s equation, the theoretical contact angles were calculated from the obtained ${Ø}_{s}$ and the WCAs on the flat films. From the Figure 12, we can see that the WCA increases with the 7F-MAP-POSS content increases and all the measured values of the copolymers containing 7F-MAP-POSS were higher than the calculated ones. Notably, the highest WCA was measured to be 153° with the biggest difference compared to the theoretical value when the fluorinated POSS content increases up to 25 wt %. This is because the convex structures were mountain-like shapes with uneven distribution in the high direction. Thus, the real contact area is smaller than the calculated value from the image above.

**Figure 13:**
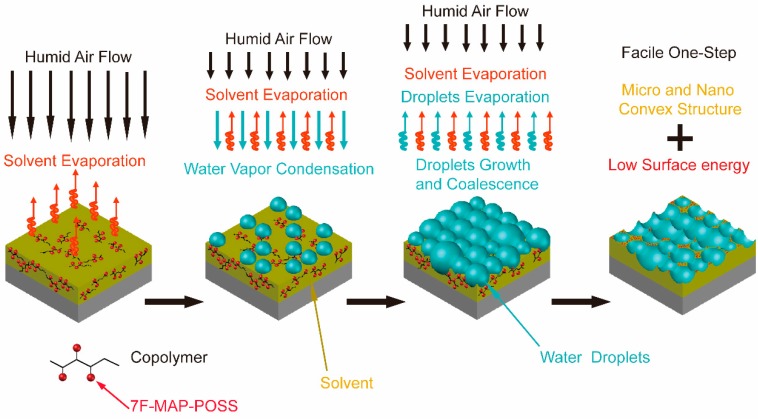
The formation mechanism of the superhydrophobic surface preparation through the one-step breath figures (BF) method.

Although the procedure is simple, the formation mechanism of BF arrays is very complicated. According to the above analysis, the formation mechanism of micro-nano structure with low surface energy using the one step BF method was deduced and illustrated by Figure 13. Above all, the as-prepared copolymer dissolved in chloroform is cast onto a substrate and then exposed to humid air flow. The temperature decreases quickly due to the endothermic evaporation of chloroform. In the following period, water condenses in the form of heterogeneous droplets on the partial surface. Then, compared with a tight and organized arrangement formed in the traditional BF process, disordered water droplets array was formed and resulted in the attenuation of the water droplets. Meanwhile, the condensed droplet can sink into the solution. The migration and enrichment of PFHEMA and 7F-MAP-POSS onto the surface occurred in the whole process. After the chloroform and water droplets completely evaporated, the micro-nano structures with low surface energy were obtained.

### 3.3. Anticorrosive Performance

**Figure 14:**
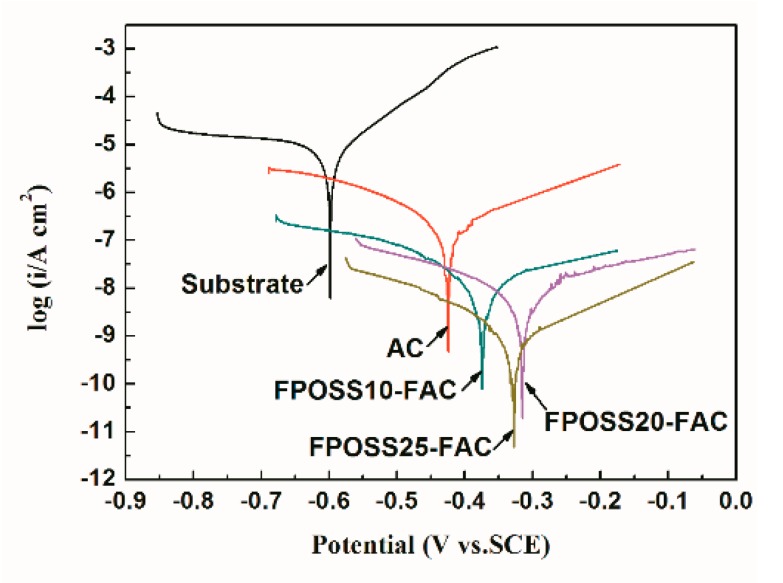
The polarization curves of substrate and all coating systems after the immersion of 7 days.

Tafel curves and EIS data are two main methods for the evaluation of material corrosion situation [45]. For comparison, acrylic resin films without the 7F-MAP-POSS were prepared by casting commercial acrylic resin (YP2226, Shanghai yoo-pont chemical industry Co., LTD., Shanghai, China) solution with the same volume and concentration onto a clean Q235 iron plate under atmosphere condition. After 7 d immersion, the Tafel curves and electrochemical parameters of the substrate, commercial acrylic resin film (AC) and as-prepared specimens containing 7F-MAP-POSS (FPOSS10-FAC, FPOSS20-FAC and FPOSS25-FAC) were depicted in Figure 14 and Table 2, respectively.

Obviously, the Tafel curves of these coating systems shifted to the positive direction compared with Q235 steel, suggesting that the coatings as a shield could effectively restrain the corrosion of Q235 steel [46]. In the meantime, with the exception of the base substrate, the commercial acrylic coating presented the lowest E_corr_ (−0.424 V) and the highest i_corr_ (4.43 × 10^−7^ A cm^−2^), which indicated the fluorinated coating could obviously enhance the E_corr_ and reduce the i_corr_ values. Especially, for the FPOSS25-FAC specimen, the i_corr_ value was 1–2 orders of magnitude lower than that of other coatings. This could be explained by the highest water repellency due to the formation of micro and nanostructures. Furthermore, the inhibition efficiency (IE) was consistent with the evolution of i_corr_ value, the result of which were 96.44% (AC), 99.22% (FPOSS10-FAC), 99.83% (FPOSS20-FAC) and 99.98% (FPOSS25-FAC), suggesting the addition of 25 wt % 7F-MAP-POSS produced the best shielding effect for Q235 substrate.

**Figure 15:**
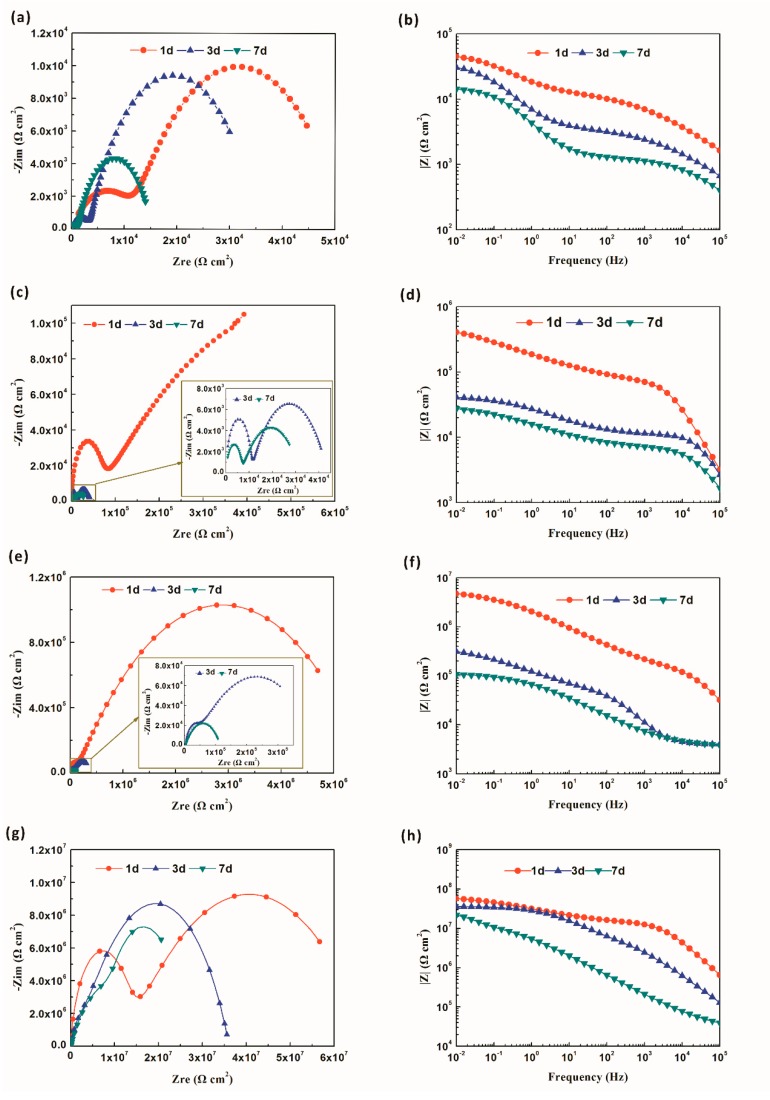
The Nyquist and Bode plots of commercial acrylic resin (AC) (**a**,**b**), FPOSS10-FAC (**c**,**d**), FPOSS20-FAC (**e**,**f**) and FPOSS25-FAC (**g**,**h**) at various immersion times under 3.5% NaCl solution.

The EIS data of Q235 substrate and as-prepared specimens was shown in Figure 15. For Nyquist image, two capacitive loops were observed for all specimens after immersion for 1 d (Figure 15a,c,e,g). Meanwhile, the capacitive loop of all specimens displayed a contractive trend as the increase of immersion time, indicating a decline in corrosion resistance. However, the radii of the capacitive loop were increased by the addition of 7F-MAP-POSS and the improvement degree was the largest for FPOSS25-FAC coating. For Bode plot, the impedance modulus at low frequency |Z|_0.01_ Hz was broadly used to evaluate the barrier ability of coating [47]. The higher the |Z|_0.01_ Hz is, the more outstanding the anticorrosion ability is [48]. It can be seen that the |Z|_0.01_ Hz decreased with the increased of immersion time for all coatings, which was because of the permeation of corrosion medium. The |Z|_0.01_ Hz of the commercial acrylic coating was about 4.51 × 10^4^ Ω cm^2^ during the initial stage of immersion (24 h) and then decreased to 1.43 × 10^4^ Ω cm^2^ after 7 d immersion (Figure 15b). For 7F-MAP-POSS-containing coatings, the initial |Z|_0.01_ Hz of FPOSS10-FAC, FPOSS20-FAC and FPOSS25-FAC specimens were 4.07 × 10^5^, 4.74 × 10^6^ and 5.71 × 10^7^ Ω cm^2^, respectively, implying a significant protective function for bare substrate (Figure 15e,h,k). After 7 d immersion, the |Z|_0.01_ Hz of FPOSS10-FAC, FPOSS20-FAC and FPOSS25-FAC specimens was reduced to 2.75 × 10^5^, 1.07 × 10^6^ and 2.16 × 10^7^ Ω cm^2^, respectively. In addition, the |Z|_0.01_ Hz of FPOSS25-FAC specimen with the super-hydrophobic surface at different immersion time was evidently higher than other coatings, manifesting the best anticorrosion property.

**Figure 16:**
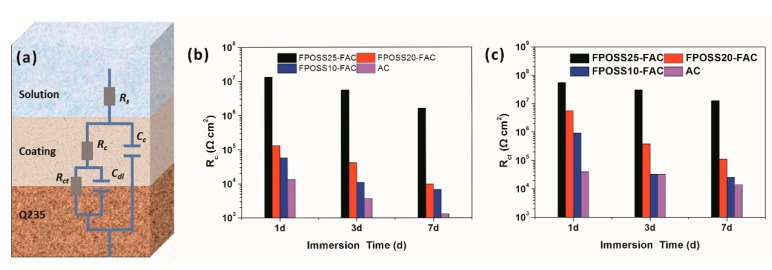
Electrical equivalent circuit models (**a**) and R_c_ (**b**) and R_ct_ (**c**) of the coating systems at various immersion times under 3.5% NaCl solution.

For further investigations, combining the EIS data with its suited electrical equivalent circuits, we could compare the electrochemical behaviour of various coatings. These components, such as R_s_, R_c_, R_ct_, C_c_ and C_dl_, represent the solution resistance, pore resistance, charge transfer resistance, coating capacitance and double-layer capacitance, respectively (Figure 16a). Among them, the R_c_ value was often chosen to assess the barrier effect of the specimen. R_ct_ value was selected to reflect the corrosion degree between coating and substrate [49,50]. By calculation, the R_c_ and R_ct_ values for different coating systems in 3.5% NaCl solution were shown in Figure 16b,c. In terms of R_c_ value, the commercial acrylic coating presented the lowest R_c_ value than other coating systems and its R_c_ decreased from 1.30 × 10^5^ to 1.28 × 10^3^ Ω cm2 after the immersion of 7 days. FPOSS25-FAC coating with superhydrophobic surface exhibited the highest R_c_ value of 1.32 × 10^7^ Ω cm^2^ at the initial immersion and then maintained at 1.62 × 10^6^ Ω cm^2^ after 7 days immersion. For R_ct_ value, the evolution of R_ct_ values for different coating systems showed a similar tendency as R. The barrier ability of the coating system was sharply enhanced and the corrosion rate of Q235 steel was significantly reduced with the addition of 7F-MAP-POSS, especially forFPOSS25-FAC coating system.

## 4. Conclusions

A superhydrophobic coating was successfully fabricated by BF method using an acrylic copolymer containing fluorinated POSS (P(MMA-BA-AA-PFHEMA-7F-MAP-POSS)). The enhanced hydrophobicity of the copolymer synchronously promoted the coalescence of the droplet to form the micro-nano convex structure and the enrichment of fluorinated groups on the surface to reduce the surface energy. The as-prepared superhydrophobic coating owned the lowest the lowest i_corr_ (2.01 × 10^−9^ A cm^−2^), the highest E_corr_ (−0.320 V), the highest |Z|_0.01_ Hz (2.16 × 10^7^ Ω cm^2^), the highest R_c_ 1.62 × 10^6^ Ω cm^2^ and the highest R_ct_ (1.26 × 10^7^ Ω cm^2^) after immersion of 7 d in 3.5% NaCl solution. In summary, this experimental study proposed a facile one-step breath method to prepare the superhydrophobic coating using a fluorinated POSS acrylic copolymer, which has a potential application in the anticorrosion area.

## Figures and Tables

**Table 1:** Molecular Weights and distribution of acrylate polymer with different F-POSS contents.

Sample	FPOSS (wt %)	*M*n (g/mol)	*M*w (g/mol)	PDI (*M*w/*M*n)
FAC	0	14,293	28,215	1.974
FPOSS10-FAC	10	15,435	30,669	1.987
FPOSS20-FAC	20	16,106	31,378	1.945
FPOSS25-FAC	25	15,640	29,415	1.881

**Table 2:** The electrochemical parameters of the substrate and the coating systems.

Sample	E_corr_ (V)	i_corr_ (A cm^−2^)	IE%
Substrate	–0.598	1.25 × 10^−5^	-
AC	–0.424	4.43 × 10^−7^	96.44
FPOSS10-FAC	–0.371	7.20 × 10^−8^	99.42
FPOSS20-FAC	–0.318	2.11 × 10^−8^	99.83
FPOSS25-FAC	–0.320	2.01 × 10^−9^	99.98
